# Test–Retest Reliability of a Questionnaire on Motives for Physical Activity among Adolescents

**DOI:** 10.3390/ijerph17207551

**Published:** 2020-10-17

**Authors:** Jaroslava Kopcakova, Zuzana Dankulincova Veselska, Michal Kalman, Daniela Filakovska Bobakova, Dagmar Sigmundova, Andrea Madarasova Geckova, Daniel Klein, Jitse P. van Dijk, Sijmen A. Reijneveld

**Affiliations:** 1 Department of Health Psychology and Research Methodology, Faculty of Medicine, P.J. Safarik University in Kosice, Tr. SNP 1, 040 01 Kosice, Slovakia; zuzana.veselska@upjs.sk (Z.D.V.); daniela.filakovska@upjs.sk (D.F.B.); andrea.geckova@upjs.sk (A.M.G.); 2 Graduate School Kosice Institute for Society & Health, P.J. Safarik University in Kosice, Tr. SNP 1, 040 01 Kosice, Slovakia; j.p.van.dijk@umcg.nl; 3 Institute of Active Lifestyle, Faculty of Physical Culture, Palacky University in Olomouc, Tr. Miru 117, 771 11 Olomouc, Czech Republic; michal.kalman@upol.cz (M.K.); dagmar.sigmundova@upol.cz (D.S.); 4 Olomouc University Social Health Institute, Palacky University in Olomouc, Univerzitní 22, 771 11 Olomouc, Czech Republic; 5 Institute of Mathematics, Faculty of Natural Sciences, P.J. Safarik University in Kosice, Jesenná 5, 040 01 Kosice, Slovakia; daniel.klein@upjs.sk; 6 Department of Community & Occupational Medicine, University Medical Center Groningen, University of Groningen, A. Deusinglaan 1, 9713 AV Groningen, The Netherlands; s.a.reijneveld@umcg.nl

**Keywords:** motivation, health-related behavior, active living, Health Behavior in School-Aged Children (HBSC) study, adolescence, gender

## Abstract

The aim of this study was to investigate the test–retest reliability of the motives for undertaking physical activity (PA) items from the Health Behavior in School-Aged Children (HBSC) study questionnaire among Slovak and Czech adolescents and to determine whether this reliability differs by gender, age group and country. We obtained data from 580 students aged 11 and 15 years old (51.2% boys) who participated in a test and retest study with a four-week interval in 2013 via the Health Behavior in School-Aged Children cross-sectional study in the Czech Republic and Slovakia. We estimated the test–retest reliability of all 13 dichotomized motives by using Intraclass Correlation Coefficients (ICC) and Cohen’s Kappa statistics, for continuous and dichotomized motives, respectively. Test–retest reliability showed moderate agreement for nine motives (ICC from 0.41 to 0.60) and fair agreement for four motives (ICC from 0.33 to 0.40). Kappa statistics were similarly moderate to large (0.33 to 0.61), except for three motives with small or trivial correlations. The motives “To improve my health” and “To enjoy the feeling of using my body” had consistently low Kappas and correlations. Overall, the results of this study suggest that most questions on motives for PA on the HBSC questionnaire have acceptable test–retest characteristics for use among adolescents.

## 1. Introduction

Physical activity (PA) is associated with adolescent healthiness and a low amount of PA is conducive of poor health outcomes and obesity during adolescence [1,2,3,4,5]. As suggested by several studies, one of the potential pathways leading to an increase in levels of PA is through motives for PA [6,7,8,9]. Accordingly, evidence-based development of national-specific strategies for public health and active living should be based on better consideration of motives for PA [7,10,11], and in addition this may increase the effects of interventions.

Several studies suggested that potential increase in level of PA is associated with specific motives for PA [7,9,12], e.g., the Goal Contents Theory, which belongs to the Self-Determination Theory field, distinguishes between specific intrinsic (such as personal growth, neighbourhood, close relationships) and extrinsic goals (such as financial success, popularity, appearance) and their impact on motivation for PA [12]. It is already known that the motives for PA are not the only determining factor of PA, but rather there are a number of interrelated factors. Moreover, several other, newer and more comprehensive approaches also address the role of motives for PA, e.g., the socio-ecological model of Sallis et al. [13,14], the Canadian 24-h movement guidelines for children and youth of Trembley et al. [15] and theories related to sense of coherence [16,17].

Motives for PA tend to differ by gender, age and geographic region [7,8,9,10,18,19]. Boys were reported as having higher rates of achievement motivation than girls, but lower rates of social and health motives than girls [7]; social and health motives were reported more frequently in older adolescents than in younger, while achievement motives were reported more frequently in older adolescent boys and in younger adolescent girls [10].

Some instruments exist for measuring motives for undertaking PA, but we know little about their stability over time, which is necessary for a valid assessment of behavioral patterns. To the best of our knowledge, only Ojala et al. [20] have investigated the test–retest reliability and validity of motives for PA items among Scandinavian students and they found that the test–retest stability was acceptable for most of the motives for PA. However, it might not be possible to generalize findings from Northern or Western Europe to Eastern Europe without caution and verification [8]. Motives for PA differ across European countries and they seem to be dependent on geographic region [8] with respect to different cultural and social standards of countries. Programs and efforts to increase motivation for PA in childhood and adolescence need to determine which tools for measuring the motives for PA are effective and beneficial for the particular population being targeted.

To our knowledge, no previous study has been devoted to the test–retest reliability of adolescent motives for PA in the Health Behavior in School-Aged Children (HBSC) survey. Questions on motives for physical activity were already used in the 1985/86 HBSC survey, as 11-item questionnaire of motivation for sports activity; in the 2005/06 HBSC survey, two questions were added. As far as we know, the test–retest reliability of the 13-item questionnaire on motives for physical activity has not been addressed before. The HBSC study provided a request for validation and in 2013 we tested selected items of the HBSC study by the test–retest study. Therefore, the aim of this study was to investigate the test–retest reliability of the motives for undertaking PA items from the HBSC study questionnaire among Slovak and Czech adolescents and to determine whether this reliability differs by gender and age group (11- and 15-year-olds) and country.

## 2. Materials and Methods

### 2.1. Study Design

This test–retest study is based on the international HBSC cross-sectional study and is consistent with its methodology. HBSC is carried out in collaboration with the World Health Organization (WHO) every four years and focuses on the health and health-related behavior of 11-, 13- and 15-year-old school children in their social context. More detailed information about the HBSC methodology can be found in Roberts et al. [21], and in these test–retest studies [22,23,24].

### 2.2. Setting, Participants and Study Size

The sample for this study came from schools in Slovakia and the Czech Republic. We contacted 12 larger and smaller primary schools located in rural as well as in urban areas in the Olomouc and Pardubice regions, Czech Republic (7 schools), and the Kosice region, Slovakia (5 schools) in November and December 2013; these regions represent the full range of adolescents in these two countries. Schools were chosen randomly from a list of schools in the Olomouc and Pardubice regions, Czech Republic, and the Kosice region, Slovakia. Inclusion criteria were the affiliation to a primary school in the Olomouc region (Czech Republic), Pardubice region (Czech Republic) or Kosice region (Slovakia), grade (5th and 9th grade) and the cognitive skills to complete the questionnaire. Exclusion criteria were primary schools educating adolescents with a special need, disagreement of parents/adolescents for study participation and inability to complete the questionnaire based on cognitive skills of the adolescents. All contacted schools agreed to participate. Questionnaires were administered in the 5th and 9th grades by trained research assistants in the absence of a teacher during regular class time. In the first part of the data collection (Test) we obtained data from 419 adolescents in the Czech Republic (response rate: 83.2%) and 259 adolescents in Slovakia (response rate: 74.1%). Non-response was primarily due to illness and parental disapproval of the participation of their children.

The second part of the data collection (Retest) was conducted four weeks after the first part. In general, it is recommended to take a period of 1–4 weeks with the goal to examine test–retest reliability of items [25]. The period between test and retest has to be sufficiently long to avoid the retention of previously given answers to questions and at the same time sufficiently short to eliminate changes in lifestyle of respondents [23]. In our study, we decided to choose a four-week period. This period is widely used also in other test–retest studies [23,26,27]. In the retest, we obtained data from 353 adolescents in Czech Republic (66 dropped out, 15.7%) and 227 adolescents in Slovakia (32 dropped out, 12.3%) who also participated in the first part of the data collection (Test). The final sample consisted of 353 Czech (51.9% boys) and 227 Slovak (52.9% boys) primary school pupils, grades five and nine.

Adolescents who participated only in the test study (the first part of the data collection) and then dropped out did not differ with statistical significance from the other respondents concerning employment status, parental education, gender and grade.

All subjects gave their informed consent for inclusion before they participated in the study. The schools in the Czech Republic had general permission granted at the beginning of the school year by all parents. Parents in Slovakia were informed about the study via the school administration and could opt out if they disagreed with it. Participation in the study was fully voluntary and anonymous, with no explicit incentives provided for participation in either country.

The study was conducted in accordance with the Declaration of Helsinki, and the protocol was approved in Czech Republic on 15 May 2012 by the Ethics Committee of the Faculty of Physical Culture, Palacky University in Olomouc under the project GACR–excellence. The protocol in Slovakia was approved on 18 June 2012 by the Ethics Committee of the Medical Faculty at the P.J. Safarik University in Kosice (No: 9/2012) under the project APVV 0032-11.

### 2.3. Variables and Data Measurement

Demographic data (age, gender) were collected using the single questions used and validated in the Health Behavior in School-Aged Children (HBSC) surveys [2,28].

**Figure 1:**
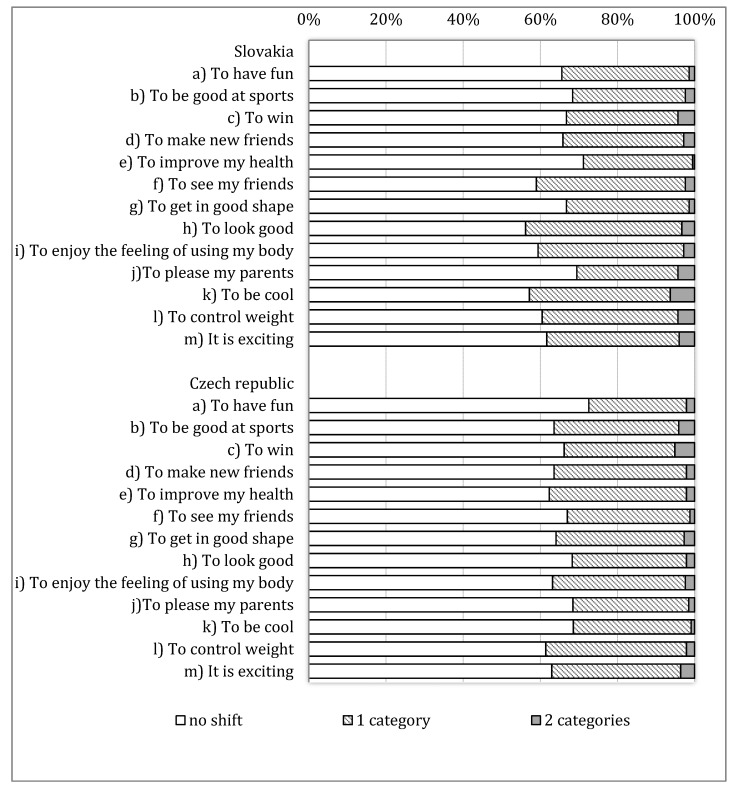
Percentage of test–retest response shifts in motives for physical activity items counted separately for Slovakia and Czech Republic.

The motives for PA were assessed using 13 items from the HBSC study examining why young people undertake leisure time PA. The question was first used as part of an optional PA package in the 1985/86 HBSC survey and the results about 11 sub-items on motivation for sports activity only from adolescents in Finland, Norway and Sweden; they were published by Wold and Kannas [29]. From the 2005/06 HBSC onward, this measure was adapted to assess motivations for all PA and the scale was broadened to include all PA and two items were added, specifically the items: “to control my weight” and “it is exciting” [8]. Since this HBSC data collection, this 13-item scale on measuring motives for PA is used. The question reads as follows: “Here is a list of reasons that some young people give for taking part in PA in their free time. For each motive please tick how important it is for you, with as answers (1) very important; (2) fairly important; (3) not important”. Respondents reply for 13 motives for PA (as can be seen in Figure 1). Further, we dichotomized all of the items by combining (1) very important and (2) fairly important vs. (3) not important.

### 2.4. Bias

We provided a maximum effort to prevent potential sources of bias in our study. The risk of selection bias was small as shown by the analyses of loss to follow-up. As our study regards the reliability of a self-report questionnaire, we cannot exclude effects of information bias including an effect of social desirability. However, this will then affect the full questionnaire as we studied.

### 2.5. Statistical Analyses

In the first step we computed frequencies of the background characteristics. Next, we assessed the proportion of respondents who answered a question identically or shifted their response by one or two categories in the test and retest. Third, we used Intraclass Correlation Coefficients (ICC) to estimate the test–retest reliability of all selected items for the whole sample and stratified by gender, age group and country. In the final step, we computed Cohen’s Kappa coefficients with dichotomized variables for the whole sample and stratified by gender, age group and country.

According to Landis and Koch’s subjective guidelines [30], the strength of test–retest agreement for an ICC greater than 0.81 is considered to be almost perfect agreement; 0.61 to 0.80 is considered to be substantial agreement; 0.41 to 0.60 is considered to be moderate agreement; 0.21 to 0.40 is considered to be fair agreement; and an ICC below 0.20 is considered to be poor. Regarding Cohen’s Kappa statistics, correlation coefficients greater than 0.5 are considered to be large, 0.3–0.5 moderate, 0.1–0.3 small and less than 0.1 are considered to be trivial [31]. All data were analyzed using IBM SPSS 20 for Windows (IBM Corp. Released 2011, Armonk, NY, USA). We used power analyses to justify group sizes to ensure they had enough power to detect among-group differences.

## 3. Results

### 3.1. Participants and Descriptive Data

The background characteristics (prevalence rates) of the study sample in the test and retest data collection can be seen in Table 1.

### 3.2. Main Results

The proportion of respondents who answered a question identically varied from 62% to 73% in the Czech Republic and from 56% to 71% in Slovakia (Figure 1).

Table 2 shows the ICCs for the HBSC items regarding motives for PA by gender, age group and country. Across subgroups and motives, the ICC varied from 0.29 to 0.65, which indicates fair to moderate agreement. Test–retest reliability showed moderate agreement for nine motives (ICC from 0.41 to 0.60) and fair agreement for four motives (ICCs from 0.33 to 0.40 for “to have fun”, “to improve my health”, “to see my friends” and “to enjoy the feeling of using my body”) in the whole sample. Motives for PA tended to have greater agreement in girls than in boys. Likewise, most motives for PA tended to have greater agreement in the 15-year-old adolescents than in the 11-year-old adolescents. Agreement tended to be better for adolescents in Slovakia than for those in the Czech Republic for most of the items.

We dichotomized all 13 motives for PA according to WHO recommendations and we created binary variables of them for further analyses. Table 3 shows Cohen’s Kappa for the HBSC items regarding motives for PA by gender, age group and country. We found strong or moderate correlations between test and retest for 10 out of 13 motives for PA in the whole sample. Moreover, we also observed strong or moderate correlations between test and retest for most of the motives per stratum of gender, age group and country. Weak correlations were observed regarding two motives (“to make new friends” and “to enjoy the feeling of using my body”) and a trivial correlation in the motive “to improve my health” in the whole sample and also per gender, age group and country. Using a binary format resulted in similar findings to using a continuous format of motive variable. The only exception was the motive “To improve my health” which showed different results and trivial agreement after dichotomization. The test–retest reliability of motives for PA tended to be better in boys than in girls. Six of the motives for PA had a somewhat better reliability in 15-year-old adolescents than in 11-year-old adolescents (a–c, g, i, l). Likewise reliability tended to be better in Slovakia than in the Czech Republic for most motives for PA. We performed power analysis and this showed that the sample sizes were sufficient to detect among-group differences with sufficient power. A sample size 150 is required to achieve Intraclass Correlation Coefficients of 0.2 in case of test and retest with power of at least 80%. A sample size 185 is required to achieve Cohen’s Kappa coefficients of 0.2 while test and retest differ by 10% in marginal frequencies with power of at least 80%.

## 4. Discussion

The aim of the study was to investigate the test–retest reliability of the motives for PA items of the HBSC questionnaire in Czech and Slovak adolescents and to determine whether this reliability differs by gender, age group (11- and 15-year-olds) and country. The motives for PA items showed moderate agreement for most motives in the whole sample and also stratified by gender, age group and country. After dichotomization, we observed a moderate correlation between the test and retest in almost all examined items, exceptions being small correlations for the motives “to make new friends” and “to enjoy the feeling of using my body” and a trivial correlation for the motive “to improve my health”.

The test–retest reliability was moderate for nine motives and fair for four motives in the whole sample, and showed a better test–retest reliability in girls than in boys, and in 15-year-old adolescents than in 11-year-old adolescents. According to our knowledge, no previous study assessed the test–retest reliability of all 13 sub-items on adolescents’ motives for PA as used in the HBSC study. We therefore can only compare our findings with those on adjacent concepts. Ojala et al. [20] reported in a study on motives for exercise that test–retest reliability was acceptable for adolescents, using a similar instrument as in the HBSC study. Wold et al. [9] assessed changes in motives for PA among adolescents from 1986 to 2006. They found that adolescents in 2006 tended to report higher importance of motives for PA than adolescents of the same age 20 years earlier in Finland, Norway and Wales. Among similar measured constructs such as, e.g., motives for food choice [32], and motives of smoking [33,34], test–retest reliability was found to be acceptable.

With further similar patterns as for the ICC, we observed strong or moderate correlations between test and retest for ten out of thirteen dichotomized motives, both in the whole sample, and per stratum of gender, age group and country. Based on our results, the HBSC questionnaire on motives for PA is an acceptable instrument to measure motives for PA among adolescents, with caution that three dichotomized motives had small or trivial correlations. It is important to identify carefully motivation for PA in adolescence and determine which instruments for measuring motives for PA are beneficial and effective for them.

Nevertheless, it is important to understand the possible determinants associated with motives for PA and PA in the period of adolescence which might be crucial for the next prevention and promotion among adolescents. Previous studies found that a motivation is a typical personal characteristic and it may be crucial for explaining that some people are sufficiently physically active in their free time [35]. Moreover, the amount of PA and the motives for PA differ highly by gender and age [8,10,35,36] and motives for PA thus vary by several factors. Our findings are in line with previous studies based on the self-determination theory [12]. In addition, previous studies showed that other extrinsic factors also had an important role, e.g., neighbourhood and environment [37], classmate and teacher support during physical education lessons [38,39] and support by family and peers [40,41].

The main strengths of this study are its large sample size, representative dataset of adolescents from two countries and collection of data according to a standardized protocol. The test–retest reliability—answering twice the same questions within a certain timeframe—can be influenced by many factors, e.g., the interpretation or understanding of a question, such as the familiarity with the content, the complexity and ambiguity of an item, the role of someone´s memory, and the number of response options [42,43]. Another strength and at the same time a possible limitation of our study is the period between test and retest administration (4 weeks) from two Central European countries which was sufficiently long to avoid the retention of previously given answers on questions and at the same time sufficiently short to eliminate changes in lifestyle of respondents. At the same time, it might be seen as a limitation of our study. Taking into account that some participants may have shifted their pattern of motives for PA during this period, it would be helpful if the methodology of similar test–retest studies became the same.

A limitation of our study is that test–retest reliability was only analyzed using ICC and Cohen’s Kappa per item, but not for an overall scale of motives for PA. Use of a fully consistent scale for motives for PA might add knowledge on the reliability with which this can be measured. Further, a proportional bias was not addressed in our study. In addition, this study was focused on the test–retest reliability of motives for the PA questionnaire within the HBSC study but did not address their validity; this would be an important issue for future research.

## 5. Conclusions

Motives for PA showed mostly moderate agreement and likewise mostly strong or moderate correlation after dichotomization in both genders in 11- and 15-year-old adolescents. We conclude that the HBSC questionnaire on motives for PA is with some caution an acceptable instrument to measure motives for PA among adolescents. Moreover, we recommend use continuous-level variables (ICC) responses for this HBSC questionnaire on motives for PA instead of dichotomized responses (Cohen´s Kappa coefficients). Motives for PA thus not only align with actual levels of PA but can also be measured pretty reliably. The study offers unique and interesting insights into how adolescents perceive motives for PA in the Czech and Slovak Republics. This study focused on the test–retest reliability of the selected items of motives for PA, but not for their validity. Moreover, future research and practice should focus on developing instruments that have better precision with the goal of reducing or overcoming the methodological bias of the studied data.

## Figures and Tables

**Table 1:** Demographic characteristics of the sample (Slovakia and Czech Republic, 2013).

	Test								Retest							
	Boys		Girls		11-Year-Olds		15-Year-Olds		Boys		Girls		11-Year-Olds		15-Year-Olds	
	*N*	%	*N*	%	*N*	%	*N*	%	*N*	%	*N*	%	*N*	%	*N*	%
Slovakia	134	38.2	125	38.3	132	37.5	127	39.0	120	40.4	107	37.8	114	36.9	113	41.7
Czech Republic	217	61.8	201	61.7	220	62.5	199	61.0	177	59.6	176	62.2	195	63.1	158	58.3
TOTAL	351	100.0	326	100.0	352	100.0	326	100.0	297	100.0	283	100.0	309	100.0	271	100.0

**Table 2:** Intraclass Correlation Coefficients (ICC) for HBSC items regarding motives for physical activity by gender, age group and country (Slovakia and Czech Republic, 2013).

	All (*N* = 580)		Gender				Age				Country			
			Boys (*n* = 297)		Girls (*n* = 283)		11 Years (*n* = 309)		15 Years (*n* = 271)		Slovakia (*n* = 227)		Czech (*n* = 353)	
	ICC	95% CI	ICC	95% CI	ICC	95% CI	ICC	95% CI	ICC	95% CI	ICC	95% CI	ICC	95% CI
Motives for PA														
a) To have fun	0.38 °°	0.31–0.45	0.36 °°	0.26–0.46	**0.41 °°**	0.30–0.50	0.32 °°	0.22–0.42	**0.46 °°**	0.35–0.55	0.36 °°	0.24–0.48	0.40 °°	0.31–0.49
b) To be good at sports	**0.56 °°**	0.50–0.61	**0.55 °°**	0.46–0.62	**0.54 °°**	0.45–0.62	**0.46 °°**	0.36–0.54	0.65 °°°	0.58–0.72	0.64 °°°	0.55–0.71	**0.50 °°**	0.42–0.58
c) To win	**0.59 °°**	0.54–0.65	**0.65 °°°**	0.57–0.71	**0.48 °°**	0.38–0.57	**0.58 °°**	0.49–0.65	0.61 °°°	0.53–0.68	0.64 °°°	0.55–0.71	**0.56 °°**	0.48–0.63
d) To make new friends	**0.44 °°**	0.37–0.50	**0.42 °°**	0.32–0.52	**0.45 °°**	0.35–0.54	0.38 °°	0.27–0.47	**0.46 °°**	0.36–0.55	**0.49 °°**	0.38–0.58	0.40 °°	0.30–0.48
e) To improve my health	0.33 °°	0.25–0.40	0.29 °°	0.18–0.39	0.36 °°	0.25–0.46	0.28 °°	0.17–0.38	0.38 °°	0.28–0.48	0.40 °°	0.28–0.50	0.28 °°	0.18–0.38
f) To see my friends	0.40 °°	0.33–0.47	0.38 °°	0.27–0.48	**0.43 °°**	0.33–0.52	**0.44 °°**	0.34–0.53	0.36 °°	0.25–0.46	0.30 °°	0.17–0.42	**0.47 °°**	0.38–0.55
g) To get in good shape	**0.50 °°**	0.44–0.56	**0.45 °°**	0.35–0.54	**0.56 °°**	0.47–0.63	**0.45 °°**	0.36–0.54	**0.56 °°**	0.47–0.64	**0.54 °°**	0.43–0.63	**0.48 °°**	0.39–0.56
h) To look good	**0.58 °°**	0.52–0.64	**0.60 °°**	0.52–0.67	**0.57 °°**	0.48–0.64	**0.60 °°**	0.52–0.67	**0.54 °°**	0.45–0.62	**0.51 °°**	0.40–0.61	0.63 °°°	0.56–0.69
i) To enjoy the feeling of using my body	0.39 °°	0.32–0.46	0.35 °°	0.25–0.45	**0.41 °°**	0.31–0.51	0.35 °°	0.24–0.45	**0.42 °°**	0.31–0.51	0.30 °°	0.17–0.42	**0.44 °°**	0.35–0.52
j) To please my parents	**0.56 °°**	0.50–0.62	**0.50 °°**	0.40–0.58	0.62 °°°	0.54–0.69	**0.45 °°**	0.36–0.54	**0.51 °°**	0.41–0.59	**0.57 °°**	0.47–0.66	**0.55 °°**	0.48–0.62
k) To be cool	**0.60 °°**	0.54–0.65	**0.59 °°**	0.51–0.66	0.61 °°°	0.52–0.68	**0.58 °°**	0.50–0.66	0.61 °°°	0.53–0.68	**0.54 °°**	0.44–0.63	0.36 °°	0.27–0.45
l) To control weight	**0.46 °°**	0.39–0.52	**0.43 °°**	0.32–0.52	**0.49 °°**	0.39–0.57	**0.41 °°**	0.30–0.50	**0.50 °°**	0.40–0.59	**0.44 °°**	0.32–0.54	**0.47 °°**	0.38–0.55
m) It is exciting	**0.54 °°**	0.48–0.60	**0.58 °°**	0.49–0.65	**0.49 °°**	0.39–0.57	**0.54 °°**	0.45–0.62	**0.54 °°**	0.45–0.62	**0.55 °°**	0.45–0.64	**0.52 °°**	0.44–0.60

°°° ICC value ˃ 0.81—perfect agreement; °°° 0.61–0.80—substantial agreement; **°° 0.41–0.60—moderate agreement**; °° 0.21–0.40—fair agreement; ° ICC value < 0.20—poor agreement (Landis, Koch, 1977).

**Table 3:** Cohen’s Kappa coefficients with dichotomized variables for Health Behavior in School-Aged Children (HBSC) items regarding motives for physical activity by gender, age group and country (Slovakia and Czech Republic, 2013).

Items	All (*N* = 580)	Gender		Age		Country	
		Boys (*n* = 297)	Girls (*n* = 283)	11 Years (*n* = 309)	15 Years (*n* = 271)	Slovakia(*n* = 227)	Czech(*n* = 353)
	Cohen’s Kappa	Cohen’s Kappa	Cohen’s Kappa	Cohen’s Kappa	Cohen’s Kappa	Cohen’s Kappa	Cohen’s Kappa
Motives for PA							
a) To have fun	0.36 **	0.29 **	0.41 **	0.21 **	0.59 **	0.45 **	0.27 **
b) To be good at sports	0.50 **	0.50 **	0.48 **	0.41 **	0.57 **	0.66 **	0.39 **
c) To win	0.56 **	0.67 **	0.44 **	0.54 **	0.58 **	0.61 **	0.53 **
d) To make new friends	0.28 **	0.30 **	0.27 **	0.34 **	0.24 **	0.37 **	0.18 **
e) To improve my health	−0.03	−0.02	−0.03	−0.03	−0.02	−0.02	−0.03
f) To see my friends	0.36 **	0.41 **	0.32 **	0.40 **	0.29 **	0.28 **	0.41 **
g) To get in good shape	0.33 **	0.29 **	0.37 **	0.17 **	0.57 **	0.32 **	0.34 **
h) To look good	0.54 **	0.59 **	0.48 **	0.55 **	0.46 **	0.46 **	0.60 **
i) To enjoy the feeling of using my body	0.25 **	0.20 **	0.29 **	0.23 **	0.28 **	0.14 *	0.30 **
j) To please my parents	0.48 **	0.41 **	0.54 **	0.56 **	0.43 **	0.53 **	0.43 **
k) To be cool	0.61 **	0.60 **	0.61 **	0.65 **	0.57 **	0.47 **	0.39 **
l) To control weight	0.42 **	0.46 **	0.37 **	0.38 **	0.45 **	0.33 **	0.48 **
m) It is exciting	0.51 **	0.61 **	0.42 **	0.53 **	0.47 **	0.58 **	0.44 **

* *p* < 0.05; ** *p* < 0.01.

## References

[1] Iannotti R.J., Kogan M.D., Janssen I., Boyce W.F. (2009). Patterns of adolescent physical activity, screen-based media use, and positive and negative health indicators in the U.S. and Canada. J. Adolesc. Health.

[2] Currie C., Zanotti C., Morgan A., Currie D., de Looze M., Roberts C., Samdal O., Smith O.R.F., Barnekow V. (2012). Social Determinants of Health and Well-Being among Young People. Health Behaviour in School-Aged Children (HBSC) Study: International Report from the 2009/10 Survey.

[3] Telama R. (2009). Tracking of physical activity from childhood to adulthood: A review. Obes. Facts.

[4] Hallal P.C., Victora C.G., Azevedo R.M., Wells J.C.K. (2006). Adolescent physical activity and health. A systematic review. Sports Med..

[5] Bauman A.E., Reis R.S., Sallis J.F., Wells J.C., Loos R.J., Martin B.W. (2012). Correlates of physical activity: Why are some people physically active and others not?. Lancet.

[6] Nigg C.R. (2003). Do sport participation motivations add to the ability of the transtheoretical model to explain adolescent exercise behavior?. Int. J. Sports Med..

[7] Kopcakova J., Dankulincova Veselska Z., Madarasova Geckova A., Kalman M., van Dijk J.P., Reijneveld S.A. (2015). Do motives to undertake physical activity relate to physical activity in adolescent boys and girls?. Int. J. Environ. Res. Public Health.

[8] Iannotti R.J., Chen R., Kololo H., Petronyte G., Haug E., Roberts C. (2013). Motivations for adolescent participation in leisure time physical activity: International differences. J. Phys. Act. Health.

[9] Wold B., Littlecott H., Tynjälä J., Samdall O., Moore L., Roberts C., Kannas L., Villberg J., Aarø1 L.E. (2016). Changes from 1986 to 2006 in reasons for liking leisure-time physical activity among adolescents. Scand. J. Med. Sci. Sports.

[10] Kalman M., Madarasova Geckova A., Hamrik Z., Iannotti R., Kopcakova J., Dankulincova Veselska Z. (2015). Motives for Physical Activity among Adolescents in the Czech and Slovak Republic. Cent. Eur. J. Public Health.

[11] Jodkowska M., Mazur J., Oblacińska A. (2015). Perceived barriers to physical activity among polish adolescents. Przegla̧d Epidemiol..

[12] Ryan R.M., Deci E.L. (2000). Intrinsic and extrinsic motivations: Classic definitions and new directions. Contemp. Educ. Psychol..

[13] Sallis J.F., Cervero R.B., Ascher W., Henderson K.A., Kraft M.K., Kerr J. (2006). An ecological approach to creating active living communities. Annu. Rev. Public Health.

[14] Sallis J.F., Cerin E., Conway T.L., Adams M.A., Frank L.D., Pratt M. (2016). Physical activity in relation to urban environments in 14 cities worldwide: A cross-sectional study. Lancet.

[15] Tremblay M.S., Carson V., Chaput J.P., Connor Gorber S., Dinh T., Duggan M., Faulkner G., Gray C.E., Gruber R., Janson K. (2016). Canadian 24-Hour Movement Guidelines for Children and Youth: An Integration of Physical Activity, Sedentary Behaviour, and Sleep. Appl. Physiol. Nutr. Metab..

[16] Bronikowski M. (2010). Is sense of coherence needed to keep youth physically active?. MedSport.

[17] Oztekin C., Tezer E. (2009). The role of sense of coherence and physical activity in positive and negative affect of Turkish adolescents. Adolescence.

[18] Rodrigues F., Moutão J., Teixeira D.S., Cid L., Monteiro D. (2019). Examining exercise motives between gender, age and activity: A first-order scale analysis and measurement invariance. Curr. Psychol..

[19] Zhou L., Chlebosz K., Tower J., Morris T. (2020). An exploratory study of motives for participation in extreme sports and physical activity. J. Leis. Res..

[20] Ojala K., Vuori M., Välimaa R., Villberg J., Tynjälä J., Kannas L. (2005). Reasons for exercise inventory in a school survey: Contemplations of the inventory’s reliability and structure validity. Liik. Tieta.

[21] Roberts C., Freeman J., Samdal O., Schnor C., Looze M., Gabhainn S.N., Iannotti R., The HBSC Methods Development Group (2009). The Health Behaviour in School-aged Children (HBSC) study: Methodological developments and current tensions. Int. J. Public Health.

[22] Bosakova L., Kolarcik P., Bobakova D., Sulcova M., van Dijk J.P., Reijneveld S.A., Madarasova Geckova A. (2016). Test–retest reliability of the scale of participation in organized activities among adolescents in the Czech Republic and Slovakia. Int. J. Public Health.

[23] Bobakova D., Hamrik Z., Badura P., Sigmundova D., Nalecz H., Kalman M. (2015). Test–retest reliability of selected physical activity and sedentary behaviour HBSC items in the Czech Republic, Slovakia and Poland. Int. J. Public Health.

[24] Holubcikova J., Kudlacek M., Sirucek J., Madarasova Geckova A. (2018). Test-retest reliability of selected HBSC items measuring problem behaviour among Slovak and Czech adolescents. Cent. Eur. J. Public Health.

[25] Kurpius S.E.R., Stafford M.E. (2006). Testing and Measurement: A User-Friendly Guide.

[26] Bult M.K., Verschuren O., Gorter J.W., Jongmans M.J., Piskur B., Ketelaar M. (2010). Cross-cultural validation and psychometric evaluation of the Dutch language version of the Children’s Assessment of Participation and Enjoyment (CAPE) in children with and without physical disabilities. Clin. Rehabil..

[27] Trinh O.T., Nguyen N.D., van der Ploeg H.P., Dibley M.J., Bauman A. (2009). Test–retest repeatability and relative validity of the Global Physical Activity Questionnaire in a developing country context. J. Phys. Act Health.

[28] Inchley J., Currie D. (2016). Growing up Unequal: Gender and Socioeconomic Differences in Young People’s Health and Well-Being: Health Behaviour in School-Aged Children (HBSC) Study.

[29] Wold B., Kannas L. (1993). Sport motivation among young adolescents in Finland, Norway and Sweden. Scand. J. Med. Sci. Sports.

[30] Landis J.R., Koch G.G. (1977). The measurement of observer agreement for categorical data. Biometrics.

[31] Cohen J. (1988). Statistical Power Analysis for the Behavioral Sciences.

[32] Markovina J., Stewart-Knox B.J., Rankin A., Gibney M., de Almeida M.D.V., Fischer A., Kuznesof S.A., Poínhos R., Panzone L., Frewer L.J. (2015). Food4Me study: Validity and reliability of Food Choice Questionnaire in 9 European countries. Food Qual. Prefer..

[33] Fiala K.A., D’Abundo M.L., Marinaro L.M. (2010). Construct Validity and Reliability of College Students’ Responses to the Reasons for Smoking Scale. J. Am. Coll. Health.

[34] Boudrez H., De Bacquer D. (2012). A Dutch version of the Modified Reasons for Smoking Scale: Factorial structure, reliability and validity. J. Eval. Clin. Pract..

[35] Aaltonen S., Kujala U.M., Kaprio J. (2014). Factors behind leisure-time physical activity behavior based on Finnish twin studies: The role of genetic and environmental influences and the role of motives. BioMed. Res. Int..

[36] Inchley J., Currie D., Jewell J., Breda J., Barnekow V. (2017). Adolescent Obesity and Related Behaviours: Trends and Inequalities in the WHO European Region, 2002–2014.

[37] Bucksch J., Kopcakova J., Inchley J., Troped P.J., Sudeck G., Sigmundova D., Nalecz H., Borraccino A., Salonna F., Dankulincova Veselska Z. (2019). Associations between perceived social and physical environmental variables and physical activity and screen time among adolescents in four European countries. Int. J. Public Health.

[38] Bronikowski M., Bronikowska M., Glapa A. (2016). Do They Need Goals or Support? A Report from a Goal-Setting Intervention Using Physical Activity Monitors in Youth. Int. J. Environ. Res. Public Health.

[39] Bronikowski M., Bronikowska M., Maciaszek J., Glapa A. (2018). Maybe it is not a goal that matters: A report from a physical activity intervention in youth. J. Sports Med. Phys. Fitness.

[40] Best K., Ball K., Zarnowiecki D., Stanley R., Dollman J. (2017). In search of consistent predictors of children’s physical activity. Int. J. Environ. Res. Public Health.

[41] Bakalar P., Kopcakova J., Madarasova Geckova A. (2019). Association between potential parental and peer correlates and physical activity recommendations compliance among 13–16 years old adolescents. Acta Gymnica.

[42] Otter M.E., Mellenbergh G.J., Glopper K.D. (1995). The relation between information processing variables and test-retest stability for questionnaire items. J. Educ. Meas..

[43] Borgers N., Hox J., Sikkel D. (2004). Response Effects in Surveys on Children and Adolescents: The Effect of Number of Response Options, Negative Wording, and Neutral Mid-Point. Qual. Quant..

